# Analysis of aroma and polyphenolic compounds in Saperavi red wine vinified in Qvevri

**DOI:** 10.1002/fsn3.2556

**Published:** 2021-10-19

**Authors:** Francesca Ieri, Margherita Campo, Chiara Cassiani, Silvia Urciuoli, Ketie Jurkhadze, Annalisa Romani

**Affiliations:** ^1^ QuMAP Laboratory PIN Polo Universitario Città di Prato Prato Italy; ^2^ Department of Statistic, Informatics and Applications “G. Parenti” (DiSIA) Phytolab Laboratory University of Florence FI Italy; ^3^ University of Bari “Aldo Moro” Bari Italy; ^4^ Caucasus International University Tbilisi Georgia

**Keywords:** agro‐food quality, analytical methods, polyphenols, volatile compounds

## Abstract

The purpose of this study is to analyze and characterize a Georgian red wine from Saperavi grape, obtained in Qvevri (Georgian traditional winemaking method), by using innovative techniques for the determination of the polyphenolic content, aroma, and its correlation to the sensory characteristics. This peculiar red wine, after high‐performance liquid chromatography with diode‐array detection and mass spectrometry (HPLC‐DAD‐MS), headspace solid‐phase microextraction–gas chromatography–mass spectrometry (HS‐SPME‐GC‐MS), and HS‐SPME‐GCxGC‐MS/TOF (two‐dimensional gas chromatography) chemical characterization showed a high polyphenol content (19.6 × 10^2^ mg/L, 38.4% anthocyanins) and a wide range of volatile compounds, among which terpenes were associated with the aroma of flowers, lemongrass, and wood. Analyses were also conducted to determine the total polyphenol content correlated to antioxidant activity with the Folin–Ciocalteu spectrophotometric in vitro method (4.650 g GAE/L). In conclusion, for the first time on Saperavi wine, innovative techniques such as HPLC‐DAD‐MS, GC‐MS, and GCxGC‐MS/TOF were simultaneously applied in association with the traditional analytic techniques to perform a complete chemical characterization. These activities are part of a project about circular viticulture in the Georgian territory that will lead the production of traced quality wines and the valorization of the Georgian wine sector.

## Graphical abstract

BLURB FOR ETOC:Analysys and characterization of a Georgian red wine from Saperavi grape, obtained in Qvevri (Georgian traditional winemaking method), by using innovative techniques for the determination of the polyphenolic and key volatile compounds to produce traced quality wines and the valorisation of the Georgian wine sector.

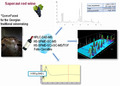

## 1 INTRODUCTION

The ancient Georgian Qvevri traditional winemaking method is considered one of the country's cultural achievements and treasures in the UNESCO ICH list, and the Georgian wine is gradually acquiring its own identity (inscribed in 2013 “8.COM” on the Representative List of the Intangible Cultural Heritage of Humanity). “Qvevri” is an oval earthenware vessel used for the Georgian traditional winemaking procedures, which include fermentation and aging of wine. (Barisashvili, 2011). Qvevri is made of a type of clay whose manufacture is made by families of craftsmen according to the traditional technology diversified from region to region. Qvevri is buried in the earth, which guarantees an optimal temperature for the aging and conservation of the wine, and its egg‐like shape helps internal processes. The basic technological process, not uniquely identified, consists of pressing the grapes; then pouring juice, grape skins, stalks, and seeds into Qvevri; and then sealing and burying it in the ground for the fermentation process. The mixture thus obtained fills Qvevri for about 80%–85%, and the whole is stirred several times a day throughout the fermentation period. When fermentation is over, Qvevri is sealed and then left to age for 5–6 months (Barisashvili, 2011; Jackson, 2008;). Now, about 530 different grape varieties are registered in Georgia's ten wine regions, some of which are widespread. Most of them are however not cultivated, mainly present in collections or in experimental vineyards. The region with the most varieties is Kakheti, where 80 different varieties are registered. Kakheti is particularly notable for its several types of highest quality wines among other Georgian regions, and especially Kakhetian wine, traditionally produced in Qvevri. The highest quality Kakhetian wine is produced in specific micro‐zones of Kakheti from grapes of Rkatsiteli, Saperavi, Kakhuri Mtsvane, Khikhvi, Kisi, and Kakhuri Mtsvivani (Glonti, 2010).

The quality of a wine and consequently the valorization of the production area depend on its numerous chemical components, whose presence or absence and amounts play an important role.

Wine aromatic profile plays a fundamental role in consumer preferences, that is, the result of the complex volatile fraction, formed by hundreds of compounds. During the aging process, wines undergo physicochemical transformations that can modulate color stability and spontaneous clarification and lead to a more complex flavor (Arfelli et al., 2007). The aroma of a wine depends on the simultaneous perception of a high number of volatile compounds, and the coupling of sensory analysis to GC‐MS analysis can provide useful indications for an adequate evaluation of the aroma. Phenolic compounds, particularly abundant in wine, represent a further contribution to the sensory and chemical quality of the final product (Baiano et al., 2014); moreover, they can produce beneficial effects on human health (Watkins Ton, 1997). HPLC‐DAD‐MS is the main technique suitable for identification and quantification of phenolic compounds in food and wines. Color is one of the main characteristics involved in the evaluation of appearance and therefore in the construction of the concept of quality by consumers, providing information about the type of wine, winemaking, and aging processes. Color can often led to the perception of other sensory characteristics as it allows one to anticipate the taste and/or olfactory properties according to the previous experience of the consumer (De Simón et al., 2008). This explains the importance of wine color in the acceptability of products (Morrot et al., 2001). The quali‐quantitative determination of the wine polyphenolic content is also useful to do reliable considerations about specific biological properties such as antioxidant, anti‐inflammatory, cardiovascular protection, and anti‐neoplastic activities, mainly correlated with the presence of polyphenols (Baur & Sinclair, 2006; Calabriso et al., 2015; Dai & Mumper, 2010; Garcia‐Alonso et al., 2009; Giovinazzo & Grieco, 2015; Paixao et al., 2007; Pandey & Rizvi, 2009).

Despite the wide availability of characterization studies regarding wines obtained from the most widespread cultivars, and despite the recognition of the quality and cultural tradition of the Saperavi grape and winemaking technique described before, at the authors’ knowledge, there are few scientific studies in the literature with an in‐depth characterization of the aromatic and polyphenolic profile of Saperavi wines.

According to the currently available results, Saperavi wines seem to have polyphenolic contents, in particular anthocyanins, comparable with more famous and studied red wines with high contents of polyphenols and anthocyanins such as Cabernet, Merlot, and Pinot Noir (Gil et al., 2012; Kekelidze et al., 2018; Kharadze et al., 2018; Mazza et al., 1999; Sergazy et al., 2019; Shalashvili et al., 2012; Wallace, 2011), whereas no data are available to evaluate its polyphenolic content and the aromatic and volatile fraction at the same time.

To the authors’ knowledge, for the first time, on Saperavi wine, techniques such as HPLC‐DAD‐MS, GC‐MS, and HS‐SPME GCxGC‐MS/TOF were simultaneously applied in association with the traditional analytic techniques to perform a complete chemical characterization. In addition, innovative headspace solid‐phase microextraction, followed by comprehensive two‐dimensional gas chromatography (HS‐SPME‐GCxGC‐TOF), was adopted for the first time, providing a volatile fingerprint of Saperavi wine.

## 2 MATERIALS AND METHODS

### 2.1 Sample

The Saperavi BATONO wine was produced in 2014 and bottled in 2015 in a winery located in Kakheti (Georgia) using the “Qvevri” vinification technique. Physicochemical parameters of the Saperavi red wine are reported in Table 1. The general Qvevri vinification method is described in the “Introduction” section, in particular the cellar of Batono company is equipped with modern machinery and devices and wine aging is carried out in earthenware vessels, based upon enologist's decision, as described in the company's website. The analyses were carried out in triplicate on three different bottles of the same harvest year. The bottles were from the same batch, and the traceability of the samples is guaranteed by the traceability of the winery's quality production system.

**TABLE 1:** Physicochemical parameters of the Saperavi red wine

Parameter	Results	Maximum acceptable limit for Georgia	Method
Density 20/20℃	0.99356		OIV‐MA‐AS2−01A
Real alcohol	14% (V/V)		OIV‐MA‐AS312−01A
Reducing sugars (a.lnv)	1.91 g/L	<4.00	OIV‐AS311−01A
Total acidity	4.7 g/L	4.0–8.0	OIV‐MA‐AS313−01
Volatile acidity	0.47 g/L	<1.20	OIV‐MA‐AS313−02
SO_2_ free	19 mg/L	<30	OIV‐MA‐AS323−04B
SO_2_ total	100 mg/L	<160	OIV‐MA‐AS323−04B
Dry extract	29.6 g/L	>20.0	OIV‐MA‐AS2−03B
Copper	0.09 mg/L	<5.00	OIV‐MA‐AS322−06
Iron	1.6 mg/L	<10.0	OIV‐MA‐AS322−05A
Lead	<0.1 mg/L	<0.300	OIV‐MA‐AS322−12
Arsenic	<0.01 mg/L	<0.200	OIV‐MA‐AS323−01A
Cadmium	<0.01 mg/L	<0.030	OIV‐MA‐AS322−10
Mercury	<0.005 mg/L	<0.005	MA−16‐AAS−72
Zinc	0.72 mg/L	<5.00	OIV‐MA‐AS322−08
Cs−137	<20.00 Bk/L	<70.00	MBИ.MH−1181–2011
Sr−90	<30.00 Bk/L	<100.00	MBИ.MH−1181–2011

### 2.2 Chemicals

All chemicals and GC standards of analytical reagent grade were from Sigma Aldrich (St. Louis, MO, USA). Ethylacetate‐D_8_; 1‐butanol‐D_10_; ethyl hexanoate‐D_11_; 5‐methyl‐hexanol; acetic acid‐D_3_; hexanoic acid‐D_11_; 3,4‐dimethylphenol; and a mixture of linear alkanes (C_10_–C_26_) in hexane for calculating linear retention indexes were used for GC analysis. Inert gasses (He and N_2_ 99.999% purity) were supplied by SOL gas company (Monza, Italy). All HPLC standards (analytical grade), DPPH (1,1‐diphenyl‐2‐picrylhydrazyl) radical, and the Folin–Ciocalteu reagent were purchased from Sigma Aldrich (St. Louis, MO, USA). All solvents (HPLC grade) and formic acid (ACS reagent) were purchased from Aldrich Company Inc. (Milwaukee, Wisconsin, USA).

### 2.3 Analysis of volatile organic compounds

Volatile organic compounds (VOCs) were analyzed by both HS‐SPME (solid‐phase microextraction)–GC‐MS and HS‐SPME‐GC × GC‐TOF analyses. Initially, some tests were carried out to optimize the quantity of sample and the exposure temperature and time, in particular varying sample dilution (2.5–5 times), absorption temperature (30℃–60℃), and absorption time (10–30 min) to check any significant influence these changes could cause to the profile. For both the analyses, SPME conditions were set as follows, according to Domizio et al., 2018: 1 ml of wine was placed into a 20‐mL screw cap vial fitted with PTFE/silicone septa, together with 2 g of NaCl, 4 ml of deionized water, and 40 μL of internal standard (ISTD) (ISTD: ethylacetate‐D_8_; 1‐butanol‐D_10_; ethyl hexanoate‐D_11_; 5‐methyl‐hexanol; acetic acid‐D_3_; hexanoic acid‐D_11_; 3,4‐dimethylphenol). The internal standard was used to normalize the analyte responses on the IS area, to minimize the instrumental error during the analysis time. After 5 min of equilibration, VOCs were absorbed exposing a 1‐cm divinylbenzene/carboxen/polydimethylsiloxane SPME fiber (DVB/CAR/PDMS by Supelco) at 60℃ for 10 min into the vial headspace under orbital shaking at 500 rpm and then immediately desorbed at 280℃ in a gas chromatograph injection port. Consistent SPME extraction conditions were ensured by a Gerstel MPS2 XL autosampler, equipped with a temperature‐controlled agitated tray (Gerstel, Mülheim an der Ruhr, Germany). Samples were analyzed in triplicate.

### 2.4 HS‐SPME‐GC‐MS analysis

The VOCs absorbed, as described in the previous paragraph, were immediately desorbed at 280℃ in the injection port of a 7890a GC system (Agilent Technologies, Santa Clara, CA, USA) operating in a splitless mode, separated by a DB InnoWAX column (0.4 μm *df* × 0.2 mm i.d., 50 m) and detected by a quadrupole mass spectrometer 5975c MSD (Agilent Technologies, Palo Alto, CA, USA) operating in EI mode at 70 eV. Initial oven temperature was set at 40℃, held for 0.5 min, then raised to 260℃ at 6℃/min, and to finish held at 260℃ for 1 min. The helium carrier gas was set at a flow rate of 1.2 ml/min. The mass spectrometer worked in the mass range 29–350 m/z, with an electron ionization of 70 eV, and the total ion current chromatograms were recorded. Compounds were tentatively identified by comparing the mass spectra of each peak with those reported in the NIST11/NISTMass Spectral Library mass spectral database, with a minimum match factor of 80%. Peak identification was then confirmed by comparing their retention index, calculated by the generalized equation (Van Den Dool & Kratz, 1963) after injecting a mixture of linear alkanes (C_10_–C_26_) in hexane in the same condition already described for sample analysis, with the literature (Chemistry WebBook). For many compounds, a positive identification was made by injecting authentic standards under the same analytical conditions. The peak areas relating to the tentatively identified compounds were normalized from Q (quantitation)‐ion, and opportune internal standard (IS), according to their chemical properties, elution order, or both. The selection of the most suitable internal standard for each analyte was done, as described by Domizio et al., 2018.

### 2.5 HS‐SPME‐GCxGC‐TOFMS analysis

GCxGC was performed by a flow modulation system consisting of an Agilent 7890B GC (Agilent Technologies, Palo Alto, CA, USA), with capillary flow modulator device for 2D separation, coupled with a time‐of‐flight mass spectrometer (TOF‐DS Markes International Ltd., Llantrisant, UK). SPME sampling was carried out at the same conditions described for mono‐dimensional GC‐MS analysis. Chromatographic separation was performed using an InnoWAX column (0.2 μm *df* × 0.18 mm i.d., 20 m) in the first dimension (1D) and an HP‐5 column (0.23 μm *df* × 0.35 mm i.d., 5 m) in the second dimension (2D). Flow modulation was performed with a modulation period of 3 s. Helium carrier gas (99.999% purity) was used at flow rates of 0.4 and 10 ml/min in first and second dimensions, respectively. The chromatographic conditions were: oven temperature program, 40℃, increased at 4℃/min to 220℃, increased at 10℃/min to 260℃ (hold 1 min); injector temperature, 260℃; split ratio 1:5. The inlet of the 2D column was maintained under vacuum by a deactivated fused silica (15 cm 0.10 mm i.d.) placed immediately before the column, after the flow modulator. TOFMS parameters: ionization, 70 eV; ion source temperature was 230℃; transfer line temperature was 280℃. A mass range of 43–500 Da with data rate of 50 Hz was used. TOF‐DS TM software, version 2.0 (Markes International Ltd.; Llantrisant, UK, 2016) was used for data acquisition. GC IMAGE version R2.5 GCGC (64 bit) software (GC IMAGE; LCC‐Lincon, Lincoln, NE, USA, 2014) was used for data processing.

Compounds were tentatively identified comparing mass spectra with those reported in mass spectral NIST11/NISTMass database; identification was confirmed by their retention index as described in 1D analysis.

### 2.6 HPLC‐DAD‐MS/TOF analysis

For HPLC‐DAD‐ESI/MS analyses, the wine samples were prepared as follows: 50.0 ml of wine was concentrated under vacuum and rinsed with 30.0 ml of water at pH 1.7 by HCOOH. Each solution was defatted with n‐hexane and extracted by liquid–liquid extraction three times, each with 20.0 ml of ethyl acetate (AcOEt). All fractions were vacuum concentrated and rinsed in 2.0 ml (AcOEt fraction) or 5.0 ml (aqueous fraction) of H_2_O/HCOOH pH 1.7. The analyses were carried out by using an HP‐1100 liquid chromatograph equipped with a DAD detector and interfaced with an Agilent TOF MS equipped with an ESI source (Agilent Corp, Santa Clara, CA, USA), as previously described (Romani et al., 2006). Quantification of the individual compounds was performed by HPLC‐DAD using five‐point regression curves built with the available standards. Curves with an r^2^>0.9998 were considered. Calibration was performed at the wavelength of the maximum UV‐vis absorbance, by applying the correction of molecular weights. In particular, the anthocyanosidic compounds were calibrated at 520 nm with malvidin 3‐glucoside (oenin); myricetin derivatives were calibrated at 350 nm with myricetin; quercetin and its derivatives were calibrated at 350 nm with quercetin 3‐glucoside; p‐coumaric acid was calibrated at 308 nm with p‐coumaric acid; hydroxycynnamic derivatives were calibrated at 330 nm with caffeic acid; vanillic acid was calibrated at 260 nm with vanillic acid; syringic acid was calibrated at 270 nm with syringic acid; resveratrol derivatives were calibrated at 308 nm with trans‐resveratrol; gallic acid, catechin, epicatechin, procyanidins, and vanillin were calibrated at 280 nm, respectively, with gallic acid, catechin hydrate, and vanillin. The determinations of the polyphenol contents were carried out in triplicate; the results are given as means, and the standard error was <5%.

### 2.7 Antiradical activity

The antiradical activity was evaluated by using the stable radical DPPH (1,1‐diphenyl‐2‐picrylhydrazyl) test. In detail, wine was diluted 1:200 and added, in a 1:1 amount, to an ethanolic solution of DPPH (0.025 mg/ml). Measurements were carried out at 517 nm with a DAD 8,453 spectrophotometer (Agilent Technologies) at time 0 and every 2 min. for the following 30 min. Antiradical activity (AR%) was calculated using the relationship: [AR% = 100 × (A0‐A30)/A0], where A0 and A30 were the absorbance of DPPH, respectively, at time 0 and after 30 min from the addition of the diluted extract (Baratto et al., 2003; Heimler et al., 2003, 2006; Huang et al., 2005; López‐Alarcón & Denicola, 2013).

### 2.8 Antioxidant activity with the Folin–Ciocalteu test

The antioxidant activity with the Folin–Ciocalteu spectrophotometric in vitro test was evaluated by using the procedure described in (Campo et al., 2016) with slight modifications. In particular, the absorbance at 725 nm was measured for a solution of the sample and the Folin–Ciocalteu reagent, after adding 20% Na_2_CO_3_ and incubating for 40 min, using a calibration curve built by measuring the absorbance of five reaction solutions containing gallic acid at different concentrations. The phenol content of the sample is expressed as GAEs (gallic acid equivalents), as [g/L of sample)].

## 3 RESULTS AND DISCUSSION

Wine is a complex alcoholic beverage containing volatile and nonvolatile components capable of interacting with aroma compounds to affect their volatility and concentration in the wine headspace and ultimately modify aroma perception and quality (Villamor & Ross, 2013). To profile and quantify volatile compounds, these can be extracted from wines using various techniques. The solid‐phase microextraction (SPME) technique is currently one of the most commonly used (Castro et al., 2008). The SPME technique is currently used to analyze volatile compounds from beverages and food (Calamai et al., 2012). According to the scientific literature, PDMS/CAR/DVB‐polydimethylsiloxane–carboxen–divinylbenzene polymer film is one of the most efficient for extracting volatile compounds from wines (Barros et al., 2012; Domizio et al., 2018; Slaghenaufi & Ugliano, 2018).

The HS‐SPME‐GC‐MS chromatogram of Saperavi wine showed a wide number of VOCs. Alcohols, esters, acids, aldehydes, and terpenes were determined. Terpenes are primary volatile compounds, called varietals. During the overripening of the grapes and during the aging of the wine, the terpenes undergo different chemical transformations which determine their decrease (Wilson et al., 1984). Terpenes are present in very small concentrations, yet they have a considerable impact on the organoleptic properties of wine (Table 2). Within this vast class of volatile components, monoterpene alcohols are those with the greatest sensory impact. In particular, linalool and geraniol are characterized by remarkably low perception thresholds. In this wine, linalool, *α*‐terpineol, *β*‐citronellol, *α*‐terpinene, *γ*‐terpinene, *p*‐cymene, and terpinolene were found (Table 3). Grapes contribute to wine aroma with numerous compounds; however, it is during fermentation that the largest number of aroma compounds are formed, mainly alcohols, acids, and esters (Schreier, 1979). Some authors attribute the basic aroma of wine to four esters (ethyl acetate, isoamyl acetate, ethyl hexanoate, and octanoate) and two alcohols, (isobutyl and isoamyl alcohol or 3‐methyl‐1‐butanol), all of which are fermentation products (Ferreira et al., 1995; Rapp & Mandery, 1986). Diethyl succinate or butanedioic acid diethyl ester was the major ester in Saperavi wine, associated with tropical fruit and floral descriptors. Isoamyl alcohol and 2‐phenyl ethanol, with whiskey, malt, and honey, rose flavor, respectively, were the major higher alcohols found in this wine.

**TABLE 2:** Terpenes founded in grapes and wines with correspondent aroma description

Descriptor	Compound
Rose	Linalool
Lily	α‐Terpineol
Citronella	β‐Citronellol
Rose	Cis‐geraniol (nerol)
Wood	α‐Terpinene
Wood	γ‐Terpinene
Citrus	p‐Cymene
Pine	Terpinolene

**TABLE 3:** Main volatile organic compounds detected by HS‐SPME‐GC‐MS in Saperavi wine. The peak areas relating to the tentatively identified compounds were normalized from Q (quantitation) ion, and opportune internal standard (A**_Q/IS_**). Data are the means of three determinations (*SD* < 5%). nd means not detected. Identification methods (ID)

Compound	A_Q/IS_	ID^a^
*Alcohols*		
Heptanol	0.04	RLI, MS
1‐Octanol	0.06	RLI, MS
2‐Methyl−1‐propanol	0.77	RLI, MS
3‐Methyl−1‐butanol	10.54	RLI, MS
1‐Hexanol	0.60	RLI, MS, RS
2‐Phenyl ethanol	6.99	RLI, MS
Benzyl alcohol	0.04	RLI, MS, RS
*Sum alcohols*	*19.04*	
*Esters*		
Nonanoic acid ethyl ester	0.01	RLI, MS
Ethyl acetate	0.17	RLI, MS, RS
Butanoic acid ethyl ester	0.12	RLI, MS
1‐Butanol, 3‐methyl‐, acetate	0,03	RLI, MS
Ethyl exanoate	0.18	RLI, MS
Ethyl lactate	0.43	RLI, MS
Octanoic acid ethyl ester	0.47	RLI, MS
Decanoic acid ethyl ester	0.08	RLI, MS
Butanedioic acid diethyl ester	0.77	RLI, MS
Acetic acid 2‐phenylethylester	0,08	RLI, MS
*Sum esters*	*2.34*	
*Acids*		
Acetic acid	0.20	RLI, MS, RS
Hexanoic acid	0.19	RLI, MS
Octanoic acid	0.58	RLI, MS
Nonanoic acid	0.22	RLI, MS
*n*‐Decanoic acid	0.30	RLI, MS
*Sum acids*	*1.49*	
*Aldehydes*		
Furfural	0.05	RLI, MS
Benzaldehyde	0.05	RLI, MS, RS
*Sum aldehydes*	*0.10*	
*Terpenes*		
Linalool	0.04	RLI, MS, RS
*α*‐Terpineol	0.02	RLI, MS, RS
*β*‐Citronellol	0.02	RLI, MS, RS
*Cis*‐geraniol (nerol)	nd	RLI, MS, RS
*α*‐Terpinene	0.19	RLI, MS, RS
*γ*‐Terpinene	0.21	RLI, MS, RS
*p‐*Cymene	0.07	RLI, MS, RS
Terpinolene	0.06	RLI, MS, RS
*Sum terpenes*	*0.61*	

^a^ Compounds were tentatively identified using reference standard (RS) analyzed under the same conditions, by comparison of the retention indices as retention linear indices (RLIs) with those from literature data and by the comparison of MS fragmentation patterns (MSs) with those reported in NIST11/NIST Mass Spectral Library mass spectral database, with a minimum match factor of 80%.

**FIGURE 1:**
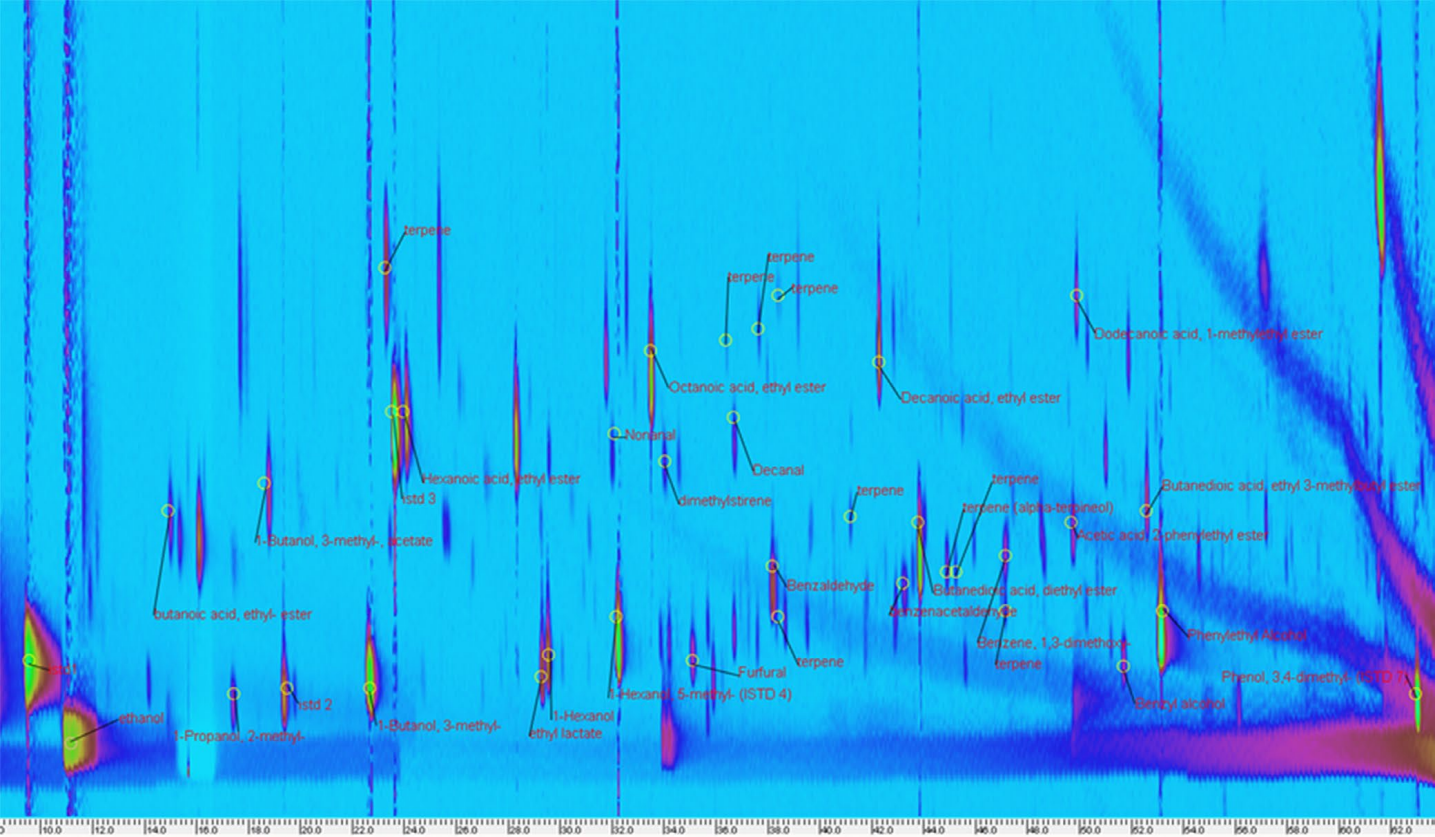
Comprehensive two‐dimensional chromatography–mass spectrometry (GC×GC‐TOF) color diagram and comprehensive template matching fingerprinting with the key identified volatile compounds of Saperavi wine

Table 3 shows the main tentatively identified volatile compounds and the key VOCs considered in this work. Compounds are ordered according to their chemical structure. HS‐SPME and GC × GC‐MS fingerprint analyses are ideal tools to analyze complex volatile matrices and offer a sensitive method for the direct comparison and chemical visualization of food volatile components. HS‐SPME‐GC × GC‐TOF‐MS analysis of the complex volatile fraction of Saperavi wine was submitted to advanced fingerprinting analysis of 2D chromatographic data (Figure 1). The use of HS‐SPME‐GC × GC‐MS analysis permitted the creation of a comprehensive template matching fingerprinting, as shown in Figure 1. This method considers, as comparative aspect, each individual 2D peak together with its MS fragmentation pattern and time coordinates and includes them in a sample template created by the analyst that can be used to directly compare plots from different samples.

Each 2D peak corresponds to a single volatile compound. The GCxGC‐TOFMS chromatogram showed around 490 peaks with an S/*N* > 500 compared with 150 peaks in 1D GC. A total of about 280 compounds were detected by GCxGC analysis of wine samples (estimated from the number of peak contours in 2D plots) after subtracting baseline peaks, corresponding to fiber blending or background interferences. The most intense peaks corresponded to butanedioic acid diethyl ester and 2‐phenyl ethanol. Dimethyl styrene (34.00, 12.79 min) and 1,3 dimethoxy benzene (47.00, 0.95 min) were identified only by GCxGC, probably owing to the co‐elution with peaks deriving from SPME fiber bleeding and/or other molecules in mono‐dimensional chromatography (34.00, 0.305 min. and 47.00, 075 min respectively). Aldehydes as nonanal, decanal, and benzeneacetaldehyde were present also in 1D chromatogram, but due to their low intensity, they were more evident in the 2D chromatogram (Figure 1).

Red wine has a particularly high content of phenolic compounds with different structures. Flavonoids are the main compound, and the main flavonoid subclasses found in wine are flavan‐3‐ol monomers (catechin and epicatechin), oligomers and polymers (proanthocyanidins or condensed tannins), anthocyanins (malvidin derivatives in particular), and flavonols (quercetin, myricetin, and kaempferol and their glycosides). Also nonflavonoid compounds are present, such as hydroxybenzoic and hydroxycinnamic acids, phenolic alcohols, stilbenes, and ellagitannins. Anthocyanins are the main phenolic compounds of red wine, whose consumption has been partially related to the “French paradox.” According to epidemiological studies, an increased consumption of anthocyanins can lower the risk of cardiovascular disease, the most common cause of mortality among men and women (Wallace, 2011). The presence of polyphenolic compounds in general is correlated with specific biological properties such as antioxidant, anti‐inflammatory, cardiovascular protection, and anti‐neoplastic activities (Baur & Sinclair 2006; Calabriso et al., 2015; Dai & Mumper 2010; Garcia‐Alonso et al., 2009; Giovinazzo & Grieco, 2015; Paixao et al., 2007; Pandey & Rizvi 2009).

The HPLC‐DAD‐MS analysis led to the identification and quantification of anthocyanins, flavonols, hydroxycinnamic acids, stilbenes, procyanidins, and other phenolic acids. The total secondary metabolites determined by HPLC/DAD was (19.6 ± 0.9) × 10^2^ mg/L, of which (7.5 ± 0.4) × 10^2^ mg/L is anthocyanins (Table 4). Malvidin 3‐glucoside was found as the predominant anthocyanin ((3.4 ± 0.2) × 10^2^ mg/L), but also the mono‐glucosides of delphinidin, petunidin, and peonidin were present in high amounts, followed by acetyl and coumaroyl glucosides of malvidin. Quercetin was found only as its glucoside and glucuronide derivatives, in a total amount of 22 ± 1 mg/L; myricetin glucoside was also found in good quantity, and kaempferol was present only in traces. High amounts of caftaric and coutaric acids and esters, respectively, of caffeic and coumaric acids with tartaric acid were present (tot (275 ± 2) × 10^2^), as well as gallic acid ((2.7 ± 0.1) × 10^2^). The total of flavan‐3‐ol derivatives, including catechin and epicatechin oligomers and polymers (procyanidins), was (4.9 ± 0.2) × 10^2^ mg/L expressed as catechin. *Trans*‐resveratrol and *cis*‐resveratrol were present as glucosides in a total amount of 82.8 ± 3.6 mg/L of wine. The HPLC/DAD/MS quali‐quantitative characterization results suggest, for the Saperavi wine under analysis, a polyphenolic composition comparable with that of other red wines with high amounts of polyphenols, anthocyanins in particular, such as Cabernet, Merlot, and Pinot Noir (Gil et al., 2012; Kekelidze et al., 2018; Kharadze et al., 2018; Mazza et al., 1999; Sergazy et al., 2019; Shalashvili et al., 2012; Wallace, 2011).

**TABLE 4:** HPLC/DAD/MS quali‐quantitative analysis of polyphenolic compounds in the Saperavi wine. Data are the means of three determinations (*SD*<5%) and are expressed as mg/L of wine. Absolute errors are reported

	**mg/L wine**
Delphinidin 3‐glucoside	72 ± 2
Cyanidin 3‐glucoside	1.51 ± 0.06
Petunidin 3‐glucoside	71 ± 3
Peonidin 3‐glucoside	71 ± 4
Malvidin 3‐glucoside	(3.4 ± 0.2) x 10^2^
Delphinidin 3‐acetylglucoside	24 ± 1
Cyanidin 3‐acetylglucoside	0.61 ± 0.03
Petunidin 3‐acetylglucoside	15.9 ± 0.7
Malvidin 3‐acetylglucoside	35 ± 2
Malvidin 3‐cumaroylglucoside	44 ± 2
Other anthocyanosides calibrated as malvidin 3‐glucoside	72 ± 3
Myricetin glucoside	5.1 ± 0.3
Quercetin glucuronide	9.7 ± 0.5
Quercetin glucoside	12.3 ± 0.6
Kaempferol and derivatives	traces
Caftaric acid	158 ± 8
Coutaric acid	117 ± 6
Caffeic acid	41 ± 2
Syringic acid	22 ± 1
*Trans*‐resveratrol glucoside	69 ± 3
*Cis*‐resveratrol glucoside	13.8 ± 0.6
Procyanidins calibrated as catechin	(4.9 ± 0.2) × 10^2^
Gallic acid and derivatives	(2.7 ± 0.1) × 10^2^
Total	(19.6 ± 0.9) × 10^2^
Total anthocyanosides	(7.5 ± 0.4) × 10^2^

The Folin–Ciocalteu assay is reported in the Office International de la Vigne et du Vin (OIV) Compendium of International Methods of Analysis of Wines and Musts (2 vol.) as an official procedure for determining the total phenolic levels in wines. The results of this in vitro assay give an evaluation of the total phenol content expressed as GAE (gallic acid equivalents), strictly correlated with the in vitro antioxidant activity. This correlation was previously demonstrated by comparisons with different assays based on electron transfer reactions (e.g., FRAP, TEAC, and ORAC) and in vitro assays on human Low Density Lipoproteins (LDL) (Huang et al., 2005; Ninfali et al., 2005; Romani et al., 2007). Saperavi wine had a high total phenolic content correlated with its antioxidant activity, measured by Folin–Ciocalteu reagent assay (4.650 g GAE/L).

The in vitro spectrophotometric assay with stable radical DPPH (1,1‐diphenyl‐2‐picrylhydrazyl) gives a measure of the radical scavenger properties of a sample, expressed as the ability to inhibit DPPH activity correlated with its absorbance at 517 nm (maximum wavelength of stable radical UV‐vis absorbance). For the Saperavi wine under analysis, percentage antiradical activity (AR%) was evaluated after 30 min of incubation of a solution 1:1 with DPPH (see “Materials and Methods section). The wine was diluted 1:200 to obtain valid kinetics, and the results indicated an anti‐free radical activity after 30 min of AR% = 74.7% for the 1:200 dilution of the wine.

## 4 CONCLUSIONS

Now, about 530 different grape varieties are registered in Georgia's ten wine regions, some of which are widespread, and the Saperavi cultivar is the most common red variety, vinified with the Georgian Qvevri traditional winemaking method. The characterization of phenolic and aromatic compounds of the traditional wine cultivar Saperavi by spectrophotometric and spectrometric techniques led the production of traced quality wines and the valorization of the Georgian territory and wine sector. In this work, the characterization of phenolic and aromatic compounds in a Georgian red wine, made from grapes of the endemic variety Saperavi, was obtained by both HPLC‐DAD‐MS and HS‐SPME‐GC‐MS analyses. To the authors’ knowledge, this study is the first one on the use of HS‐SPME‐GCxGC‐TOF to study more in deep the characterization of the volatile fraction of a Saperavi wine. In addition, in vitro antioxidant and antiradical activities were evaluated using the assays with Folin–Ciocalteu reactive and DPPH∙stable radical. This allows obtaining for the first time a complete chemical characterization of the hydrosoluble polyphenolic fraction together with the volatile fraction and the in vitro antioxidant and antiradical activities of a Saperavi wine vinified in Qvevri. The characterization of this wine is part of a project about circular viticulture in Georgian territory. The primary aim of circular viticulture is to organize a network of companies and research institutes to create a closed and innovative chain in the wine sector in order to evaluate the quality of the vineyard, monitor environmental and management parameters aimed at producing traced quality wines, and also use grapes to produce functional foods such as juices, jams, grape seed oil, and other nutraceuticals. It is also planned the exploitation of secondary raw materials and waste products for the production of organic fractions with high biological activity, which can be used in the food, cosmetic, phytotherapeutic, and agronomic sectors as well as innovative materials. The sustainable exploitation allows the final use of exhausted materials for the production of sustainable energy.

## CONFLICT OF INTEREST

The authors declare that they do not have any conflict of interest.

## ETHICAL STATEMENT

The authors declare that they do not have any conflict of interest.

This study does not involve any human or animal testing.

## Data Availability

The data that support the findings of this study are available from the corresponding author, upon reasonable request.
